# The Impact of Carnitine on Dietary Fiber and Gut Bacteria Metabolism and Their Mutual Interaction in Monogastrics

**DOI:** 10.3390/ijms19041008

**Published:** 2018-03-28

**Authors:** Abdallah Ghonimy, Dong Ming Zhang, Mohammed Hamdy Farouk, Qiuju Wang

**Affiliations:** 1 College of Animal Science and Technology, Jilin Agricultural University, Changchun 130118, China; abdomscphd@yahoo.com (A.G.); wangqiuju0439@163.com (Q.W.); 2 Tonghua Normal University, Tonghua 134000, China; 3 Department of Animal Production, Faculty of Agriculture, Al-Azhar University, Cairo 11884, Egypt

**Keywords:** absorption, butyrate, fermentation, microbial composition, propionate

## Abstract

Carnitine has vital roles in the endogenous metabolism of short chain fatty acids. It can protect and support gut microbial species, and some dietary fibers can reduce the available iron involved in the bioactivity of carnitine. There is also an antagonistic relationship between high microbial populations and carnitine bioavailability. This review shows the interactions between carnitine and gut microbial composition. It also elucidates the role of carnitine bacterial metabolism, mitochondrial function, fiber fermentability, and short chain fatty acids (SCFAs).

## 1. Introduction

Carnitine (γ-trimethylamino-β-hydroxybutyric acid) is a quaternary ammonium molecule required for the transport of long-chain fatty acids (LCFAs) into the mitochondria, where β-oxidation takes place [1]. Moreover, carnitine is involved in buffering the equilibrium between acyl-CoA and CoA [2]. Carnitine can be synthesized in mammals [3], but not in bacteria, where carnitine or its immediate precursors are imported into the cells [4]. Carnitine occurs in humans, animal products (such as meat, milk and egg), plants, and several micro-organisms [5]. Animal tissues are the main source of carnitine, especially muscles which possess about 95% of the body’s carnitine pool [6,7]. Most mammals can endogenously synthesize carnitine to provide most of their carnitine requirement; any further requirement can be supplied by their diet [5].

Carnitine can act in a direct or indirect manner with dietary fibers; this is because of the various interactions carnitine has with the gut microbiome [8]. Dietary fibers are heterogeneous and consequently have different effects on both the gut microbial community and the host animal [9,10]. The main end products of bacterial fermentation of dietary fiber are short chain fatty acids (SCFAs) [11], vitamins [10], H_2_ and CO_2_ [12]. Moreover, the intestinal microbiota can control various biological processes such as nutrient absorption, lipid and glucose homeostasis, and systemic inflammation [13]. SCFAs can improve the well-being of the host animal. For instance, butyrate is the preferred energy source for colonic epithelium cells [14]. Such an energy source can decrease the rate of formation of secondary bile acids from primary bile acids, and protect the host against colorectal cancer. Additionally, higher concentrations of primary bile acids have been observed in non-atherosclerosis patients than in atherosclerosis patients [15,16]. Moreover, propionate reduces the biosynthesis of cholesterol [17], providing protection against cardiovascular disease (CVD) [18]. Most acetate molecules are absorbed from the circulatory system by the liver, and used as an energy source. They are also used as a substrate to form cholesterol and long-chain fatty acids (LCFAs) [19,20].

Dietary carnitine is one of the main factors that can interact with dietary fibers, microbial composition [4], and related metabolites such as SCFAs [21]. The aforementioned interactions have not been well characterized. This review aims to show that carnitine is a key molecule in the metabolism of dietary fibers and gut microbial composition, and to elucidate the role of carnitine in bacterial metabolism, mitochondrial function, and fiber fermentability.

## 2. Carnitine and Bacterial Cell Functions

Carnitine and acylcarnitines (fatty acyl ester of l-carnitine) are absorbed by the lumen of the small intestine. These carnitines are transported into enterocytes by active transport, and subsequently into the circulatory system through the serosal membrane via diffusion [22,23]. Carnitine that does not get absorbed will reach the large intestine, where the microbiota is located. Carnitine that reaches the large intestine is digested by bacteria, since there are no active digestive exocrine enzyme systems that can breakdown carnitine [24]. There is no debate that bacteria can utilize the carnitine in different ways [25].

There are different mechanisms by which dietary carnitine is used as a carbon or nitrogen source. The first one is to cleave the carbon–nitrogen bond to produce trimethylamine and malic semialdehyde [4]. For example, *Acinetobacter calcoaceticus* can further degrade the carnitine into trimethylamine and malic acid, which can be used as a carbon source [26]. A similar pathway has been found in the aerobic bacterium *Serratia marcescens* [27], where the malic semialdehyde enters the tricarboxylic acid (TCA) cycle. This pathway requires two subunits of oxidoreductase that can be found in *Acinetobacter baumannii* and *Serratia marcescens* [28].

The second mechanism begins by converting carnitine to glycine betaine in the presence of adenosine triphosphate (ATP) or CoA [4,29]. Without ATP or CoA, carnitine is decarboxylated into the two compounds: trimethylaminoacetone and CO_2_ [29]. With CoA or ATP, glycine betaine is subjected to three demethylations that produce glycine which then enters the central metabolism [4]. Glycine betaine can act as either an osmolyte, carbon, nitrogen, or energy source. This decarboxylation and demethylation of carnitine has been shown in bacteria that possess the required conversion enzymes [4]. For example, Pseudomonas syringae B728a has the ability to convert carnitine to glycine betaine, because this species of bacterium possesses the required conversion genes. Brevibacterium linensconverts l-carnitine to glycine betaine, and consequently, such betaine could be utilized as a sole carbon or nitrogen source [30], whereas Pseudomonas syringae DC3000 has no carnitine catabolic operon [31,32]. *Pseudomonas aeruginosa*, *Xanthomonas translucens*, *Enterobacter* sp., *Pseudomonas putida*, *Pseudomonas fluorescens*, *Burkholderia cepacia*, *Rhizobium* sp., and *Agrobacterium* sp. can encode carnitine dehydrogenases (CDHs) that are specific for converting l-carnitine to glycine betaine [4].

*Acinetobacter calcoaceticus* and other species can utilize both d- and l-carnitines as sole carbon sources [26], since these species can produce both l-carnitine dehydrogenase (l-CDH) and d-carnitine dehydrogenase (d-CDH) [4]. *Enterobacteriaceae* such as *E. coli*, *Salmonella typhimurium*, *Proteus vulgaris* and *Proteus mirabilisis* are able to convert carnitine into γ-butyrobetaine via crotonobetaine reductase [33]. *Acinetobacter calcoaceticus* 69/V is able to utilize l-carnitine, l-*O*-acylcarnitine, and γ-butyrobetaine as sole carbon sources, d-carnitine can also be utilized as a carbon and nitrogen source, but only in the presence of l-carnitine to act as an inducer [26]. The latter pathway has been established in the species *Agrobacterium radiobacter* [34]. *Pseudomonas aeruginosa* can utilize acylcarnitines (2–16 fatty acids) as sole carbon and nitrogen sources, except for octanoylcarnitine [35]. Short-chain acylcarnitines (acetyl- and butyl-carnitine) are hydrolyzed to l-carnitine and SCFA by the esterase HocS (hydrolase of *O*-acylcarnitine, short-chains) [35]. Thus, the gut microbiota can use carnitine as a carbon source via two mechanisms.

*Rhizobium meliloti* metabolizes glycine betaine as an energy source. Such bacteria can convert glycine betaine into dimethylglycine by the action of glycine betaine transmethylase; dimethylglycine is then converted into monomethyl glycine by the action of dimethylglycine dehydrogenase. Monomethyl glycine is then converted into glycine by the action of monomethylglycine dehydrogenase, and this glycine is converted into serine by the action of serine transhydroxy methylase. Finally, serine is deaminated by serine dehydratase into pyruvate, which then enters the TCA cycle to generate ATP molecules [36].

In anaerobic conditions, where oxygen cannot act as an electron acceptor, the bacteria can use alternative electron acceptors such as sulfates, nitrates, ferric iron, carbon dioxide, and fumarate. Even when common electron acceptors are not available, some Enterobacteriaceae species (e.g., *E. coli* and Salmonella typhimurium) can use carnitine and its catabolic product (crotonobetaine) as final electron acceptors [37,38]. In aerobic conditions, Pseudomonas species, such as *Pseudomonas aeruginosa*, are able to grow on l-carnitine as their sole sources of carbon and nitrogen. Aerobic carnitine degradation starts with hydroxyl group oxidation by l-carnitine dehydrogenase to form 3-dehydrocarnitine [39]. Both Gram-positive and Gram-negative bacteria can use carnitine for different cellular functions in anaerobic or aerobic environments, because carnitine can be used as a sole nitrogen and sole carbon source, or as an electron acceptor [4]. Thus, bacteria can use carnitine for a variety of cellular functions including the former biological actions, and also to maintain the normal enteric pH. Thus, in prokaryotes, carnitine can be catabolized to act as a sole nitrogen and/or sole carbon source, or electron acceptor and osmolyte by the bacterial cell.

## 3. Carnitine and Bacterial Protection

The gut microbiota can import or synthesize compatible solutes to protect themselves against stresses resulting from salt, pressure, temperature, or changing water content. Such a solute accumulates at high concentrations in the cytoplasm, without conflicting with the normal biological processes [40]. Interestingly, carnitine is an ideal compatible solute that can be imported, or generated by many bacteria [41,42]. Carnitine can act as an osmoprotectant and/or osmolyte that can be utilized by bacteria to protect against osmotic stress [4,43].

Carnitine is transported into the cytosol of bacteria by different mechanisms, for example, *Bacillus subtilis* can import d- and l-carnitine, acetylcarnitine, crotonobetaine, c-butyrobetaine and octanoylcarnitine via the OpuC transporter, which is an ATP-binding cassette (ABC) transporter family member [44]. The expression of the OpuC carnitine uptake system is increased at low temperatures in order to protect against heat stress [4,45]. In *Listeria monocytogenes*, the OpuC carnitine transport system uses the OpuC transporter to import carnitine, which is important for protecting against bile stress [46], and maintaining the gut bacteria [47]. Another mechanism was observed in *P. aeruginosa* whereby three members of the betaine\choline\carnitine transporter (BCCT) family and a single ABC family member are produced in order to transport carnitine to the bacterial cell [48]. Furthermore, the degradation of *O*-acylcarnitines as a mechanism was observed in *B. subtilis* [35], since the degradation of *O*-acylcarnitines can provide an osmolyte to protect such microbiota against hyperosmotic stress [44]. *O*-acetylcarnitine can be hydrolyzed by HocS to generate free carnitine, which acts as an osmoprotectant [35], such as in *Bacillus subtilis* and *P. putida* [44]. These species can utilize short-chain acylcarnitines ranging from 10 to 16 carbons in length. However, *P. aeruginosa* hydrolyzes short-chain acylcarnitines ranging from 2 to 6 carbons in length by an l-enantiomer-specific hydrolase [35]. Thus, carnitine is transported into the cytosol of bacterial cells by one of three mechanisms: ABC, BCCT [49,50], or indirect degradation of *O*-acylcarnitines [48]. Interestingly, the gut bacteria not only use the imported form of carnitine, but they can also produce endocellular carnitine forms.

## 4. Microbial Carnitine Production

De novo carnitine synthesis has not been confirmed in any bacterial species [4]. By contrast, carnitine is synthesized in mammals from the amino acids lysine and methionine. The carbon backbone of carnitine is derived from lysine, and the 4-*N*-methyl groups are derived from methionine. These reactions are catalyzed by methyltransferases to release trimethyl lysine (TML). This TML is hydroxylated by trimethyl lysine dioxygenase (TMLD) to form 3-hydroxy trimethyl lysine (HTML). The later molecule is acted on by HTML aldolase to generate 4-trimethylaminobutyraldehyde (TMABA) and glycine. The TMABA is acted on by TMABA dehydrogenase (TMABA-DH) to form 4-*N*-trimethylaminobutyrate (butyrobetaine), that is finally hydroxylated by γ-butyrobetaine dioxygenase (BBD) to form carnitine [5]. However, bacteria can produce carnitine by utilizing other substrates [4], for instance, *Escherichia coli* and *Proteus* sp. can produce l-carnitine from crotonobetaine or d-carnitine using carnitinyl-CoA hydrolase and carnitine racemase [1]. *Bacillus subtilis* also utilizes acetylcarnitine to produce carnitine by the action of acyl-l-carnitine esterases. The carnitine acts as an osmolyte to protect the bacterium against cellular hyperosmotic stress [44]. Members of the family Enterobacteriaceae produce carnitine using γ-butyrobetaine as a substrate [1]. The Betaproteobacterium *Achromobacter cycloclast* and the Gammaproteobacterium *Acinetobacter calcoaceticus* have the same ability to produce carnitine from γ-butyrobetaine, and they are predicted to have similar enzymic activities to species of Enterobacteriaceae. The latter family can utilize γ-butyrobetaine hydroxylase for the biosynthesis of carnitine [4]. Therefore, gut microbiota can use different substrates to form carnitine. Further studies are needed to investigate the effect of carnitine concentration on gut bacterial composition.

## 5. The Interaction between Carnitine and the Gut Microbial Community

**Figure 1:**
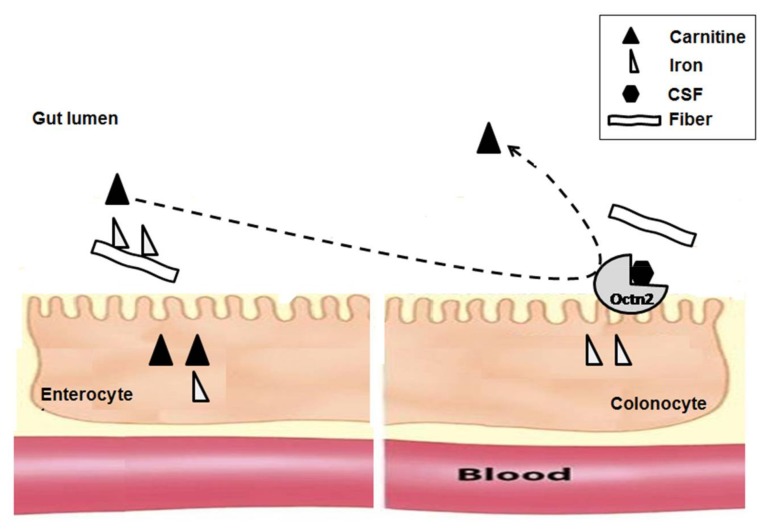
Carnitine and iron absorption in the intestines. Non-absorbed dietary carnitine reaches the large intestine (colonocytes), where bacterial sporulation-stimulating factor (CSF) competes with carnitine on the organic cation transporter novel 2 (Octn2) transporter, leading to reduced carnitine absorption. Iron conjugated to fiber is absorbed less in the small intestine (enterocytes), but is absorbed more in the large intestine, as bacterial fermentation can dissociate such conjugates.

The uptake of carnitine by the large intestine (colon) is likely to be influenced by the gut microbial population. The carnitine transporter (Octn2), which is involved in carnitine absorption, is expressed in both the small and large intestines [5]. If the colonic bacterial community reaches a high cell density, quorum-sensing systems can facilitate cell-to-cell communication within the bacterial population by producing molecules such as CSF (sporulation-stimulating factor) and LSP (lipopolysaccharide; carnitine absorption factor). Such factors can regulate gene expression in colonocytes [51]. CSF induces functions in bacterial cells and in colonocytes, since the CSF passes into bacterial cells using oligopeptide permease (Opp) [52,53]. However, CSF passes into colonocytes using organic cation transporter novel 2 (Octn2) [54]. In bacterial cells, both CSFs and ComX pheromone (a regulator protein) can work together to regulate some functions, such as genetic competence initiation, sporulation, and antibiotic synthesis [53,55]. In colonocytes, CSF passes into the cells to stimulate the expression of heat-shock inducible protein 27 (Hsp27), which protects the cells against oxidant stress [56]. However, CSF competes with carnitine for Octn2 transport, leading to an inhibition of carnitine absorption [57]. Thus, when there is a high microbial population density, carnitine absorption may be inhibited as depicted in Figure 1. Conversely, the bacterial LSP molecule activates Toll-like receptor 4 (TLR4) in colonocytes, which stimulates PPAR-γ (xenoreceptor) activity, and Octn2 gene expression, leading to an increase in the carnitine absorption rate [58,59].

## 6. Carnitine and Fatty Acid Regulation

Carnitine has a crucial role in propionic acid metabolism; this is because carnitine promotes the synthesis of propionyl-CoA carboxylase (PCC) by promoting the transcription of propionyl-CoA carboxylase A (PCCA) and propionyl-CoA carboxylase B (PCCB) genes [60]. These enzymes can convert residual SCFAs into acetyl-CoA that is used in the Krebs cycle to produce ATP molecules. Moreover, carnitine can bind to residual amounts of mitochondrial propionic acid in order to remove and excrete them into the urine [61]. Many clinical studies have suggested that an improvement in propionic acidemia disorder can occur by administering carnitine to affected individuals [21,60,62]. SCFAs may change mitochondrial function through carnitine metabolism and the Krebs cycle [21] to elicit their effects. Propionic acid induces the expression of carnitine palmitoyltransferase II (CPT-II), which is part of the carnitine transport system within the cell. Such induction can be promoted by phosphorylation of ERK kinase, leading to an upregulation of PPAR-α for CPT-II gene expression [63].

Carnitine can also activate butyrate absorption in an indirect way. As carnitine protects bacteria against abnormal salinity [64], such bacteria have an anti-inflammatory effect against cytokine TNF-α activity which inhibits butyrate transporter activity [65]. The anti-inflammatory effects of bacteria may be due to a balance between the suppression of proinflammatory mediators (TNF-α) and the induction of anti-inflammatory cytokines, which may be related to the production of SCFAs [66]. For instance, the species L. plantarum stimulates enteric butyrate absorption by modulating the sodium-coupled monocarboxylate transporter 1 (SMCT1) [65] and monocarboxylate transporter 1 (MCT1) [67]. Conversely, in inflammatory bowel disease (IBD), these transporters can be inhibited by cytokine TNF-α, and consequently butyrate uptake can be down-regulated. In addition, L. plantarum can block the inhibitory effect that cytokine TNF-α has on SMCT1 transporter expression [65].

In eukaryotes, butyrate passes the double-mitochondrial membrane to the mitochondrial matrix, where oxidation of butyrate is activated to produce acetyl-CoA, which then enters the Krebs cycle [68]. Excess acetyl-CoA inhibits butyrate oxidation, leading to a reduction in Krebs cycle capacity in colonocytes, asbutyrate provides 70% of the energy supply to the colonocytes [69]. Carnitine transfers any excess amounts of intra-mitochondrial acetyl-CoA through the carnitine acetyltransferase pathway to the cytosol [70,71], to produce fatty acids [72,73]. Therefore, carnitine can regulate energy metabolism.

Carnitine has a crucial role in fatty acid β-oxidation during the transport of activated LCFAs from the cytosol to the mitochondrial matrix, and in the modulation of the intra-mitochondrial acetyl-CoA/CoASH ratio [74,75,76]. In addition, carnitine is involved in peroxisomal β-oxidation by moving acetyl-CoA to the mitochondria for oxidation in the Krebs cycle to produce CO**_2_**, H**_2_**O [77,78], and energy [76]. Carnitine can also store energy (as acetyl groups) in the form of acetyl carnitine [6,79], and detoxify the poorly metabolized acyl groups by forming carnitine esters that are excreted out of mitochondria [80].

## 7. Fiber–Carnitine Interaction

Dietary fiber can affect carnitine homeostasis by trapping the co-factor element iron. This co-factor is necessary for carnitine biosynthesis and a deficiency results in an increase in the risk of atherosclerosis and other disorders.

Iron is an important co-factor that is required for the activity of the first enzyme in carnitine biosynthesis, as TMLD is a non-haem ferrous-iron dioxygenase. TMLD requires 2-oxoglutarate, Fe^2+^, and molecular oxygen as cofactors [81,82,83,84]. Dietary fibers can have a negative effect on iron absorption in the small intestine, as some dietary fibers can bind iron and consequently decrease the availability of iron for absorption [4]. This negative effect of fiber depends on fiber components [85], colonic fermentation [86], viscosity [87,88], pH [89,90], and fiber esterification [91]. Fiber components, such as some non-starch polysaccharides (NSP) and lignin, can form complexes with the iron and lead to further decreased iron bioavailability [85], as depicted in Figure 1. Binding occurs between iron and functional groups such as carboxyl groups (e.g., in uronic acids) or carboxyl and hydroxyl groups (e.g., in phenols) [85,89,90]. Moreover, carnitine biosynthesis requires some dietary vitamins [92] and minerals to support the intestinal microbiota [93]. There is limited research describing the effect of anti-nutritional factors or enhancers on carnitine biosynthesis cofactors and coenzymes.

A low fiber intake can stimulate carnitine breakdown by enteric microbiota, producing trimethylamine (TMA) [94]. This TMA is converted in the liver to trimethylamine-*N*-oxide (TMAO), leading to an increased risk of atherosclerosis and CVD [95].

## 8. Conclusions

Carnitine has an important role in maintaining the high fiber fermentation ability of the colonic microbiota. Colonic microbiota can use carnitine as a source of carbon, nitrogen, or as an electron acceptor. Furthermore, carnitine is utilized by the intestinal microbiota as a protective solute against different stressors. Carnitine also mediates the metabolism of SCFAs by regulating normal levels of such SCFAs in the cytosol and mitochondria, in order to provide normal energy maintenance for the host. There is an antagonistic relation between dietary iron and carnitine biosynthesis when there is a high intake of dietary fiber, and an antagonistic relation between a high intestinal bacterial population and carnitine absorption. A low dietary fiber intake can affect metabolic carnitine pathways, leading to an increase in the risks of atherosclerosis and CVD. Further investigation is required to evaluate the effects of different anti-nutritional factors on carnitine bioactivity.

## Abbreviations

ABC	ATP-binding cassette
ATP	Adenosine triphosphate
BBD	γ-Butyrobetaine dioxygenase
BCCT	Betainecholinecarnitine transporter
CDH	Carnitine dehydrogenase
CPT-II	Carnitine palmitoyltransferase II
CSF	Sporulation-stimulating factor
CVD	Cardiovascular disease
HocS	Hydrolase of *O*-acylcarnitine, short-chains
Hsp27	Heat-shock inducible protein27
HTML	3-Hydroxy trimethyl lysine
IBD	Inflammatory bowel disease
L-CDH	l-Carnitine dehydrogenase
LCFAs	Long chain fatty acids
LSP	Lipopolysaccharide
MCT1	Monocarboxylate transporter1
NSP	Non-starch polysaccharides
Octn2	Organic cation transporter novel 2
PCC	Propionyl-CoA carboxylase
PCCA	Propionyl-CoA carboxylase A
PCCB	Propionyl-CoA carboxylase B
SCFAs	Short chain fatty acids
SMCT1	Sodium coupled monocarboxylate transporter1
TLR4	Toll-like receptor 4
TMA	Trimethylamine
TML	Trimethyl lysine
TMABA	4-Trimethylaminobutyraldehyde
TMAO	Trimethylamine-*N*-oxide
TMLD	Trimethyllysine dioxygenase
